# Structure and stability of symptoms in first episode psychosis: a longitudinal network approach

**DOI:** 10.1038/s41398-021-01687-y

**Published:** 2021-11-06

**Authors:** Siân Lowri Griffiths, Samuel P. Leighton, Pavan Kumar Mallikarjun, Georgina Blake, Linda Everard, Peter B. Jones, David Fowler, Joanne Hodgekins, Tim Amos, Nick Freemantle, Vimal Sharma, Max Marshall, Paul McCrone, Swaran P. Singh, Max Birchwood, Rachel Upthegrove

**Affiliations:** 1 Institute for Mental Health, University of Birmingham, Birmingham, UK; 2 Institute of Health and Wellbeing, University of Glasgow, Glasgow, UK; 3 College of Medical and Dental Sciences, University of Birmingham, Birmingham, UK; 4 Birmingham and Solihull Mental Health Foundation Trust, Birmingham, UK; 5 Department of Psychiatry, University of Cambridge and CAMEO, Cambridge and Peterborough NHS Foundation Trust, Cambridge, UK; 6 Department of Psychology, University of Sussex, Brighton, UK; 7 Norwich Medical School, University of East Anglia, Norwich, UK; 8 Academic Unit of Psychiatry, University of Bristol, Bristol, UK; 9 Institute of Clinical Trials and Methodology, University College London, London, UK; 10 Early Intervention Service, Cheshire and Wirral NHS Foundation Trust, Liverpool, UK; 11 Lancashire Care NHS Foundation Trust, Preston, UK; 12 Institute for Life Course Development, University of Greenwich, London, UK; 13 Mental Health and Wellbeing Warwick Medical School, University of Warwick, Coventry, UK

**Keywords:** Schizophrenia, Predictive markers

## Abstract

Early psychosis is characterised by heterogeneity in illness trajectories, where outcomes remain poor for many. Understanding psychosis symptoms and their relation to illness outcomes, from a novel network perspective, may help to delineate psychopathology within early psychosis and identify pivotal targets for intervention. Using network modelling in first episode psychosis (FEP), this study aimed to identify: (a) key central and bridge symptoms most influential in symptom networks, and (b) examine the structure and stability of the networks at baseline and 12-month follow-up. Data on 1027 participants with FEP were taken from the National EDEN longitudinal study and used to create regularised partial correlation networks using the ‘EBICglasso’ algorithm for positive, negative, and depressive symptoms at baseline and at 12-months. Centrality and bridge estimations were computed using a permutation-based network comparison test. Depression featured as a central symptom in both the baseline and 12-month networks. Conceptual disorganisation, stereotyped thinking, along with hallucinations and suspiciousness featured as key bridge symptoms across the networks. The network comparison test revealed that the strength and bridge centralities did not differ significantly between the two networks (C = 0.096153; *p* = 0.22297). However, the network structure and connectedness differed significantly from baseline to follow-up (M = 0.16405, *p* = <0.0001; S = 0.74536, *p* = 0.02), with several associations between psychosis and depressive items differing significantly by 12 months. Depressive symptoms, in addition to symptoms of thought disturbance (e.g. conceptual disorganisation and stereotyped thinking), may be examples of important, under-recognized treatment targets in early psychosis, which may have the potential to lead to global symptom improvements and better recovery.

## Introduction

Psychosis is a disorder of complex psychopathology, with heterogeneous illness trajectories, particularly in the early stages [1]. Although early recognition and treatment bring substantial benefit [2], outcomes remain poor for many with first episode psychosis (FEP) [3]. Understanding how symptoms are structured, and which key symptoms play a pivotal role in maintaining psychopathology, may help to identify new treatment targets and improve outcomes.

Symptom interactions in early stages of illness are likely to be fluid and may change in strength and quality over time [4]. The interconnectedness of positive, negative and co-morbid affective symptoms in psychosis has previously been explored based on latent structures of symptomatology [5, 6], whereby symptoms may be connected via a single underlying latent variable (psychosis), and non-psychosis symptoms considered of secondary importance [6]. However, it is also suggested that the flame of positive symptoms is driven by affective dysfunction in the early years, and primary negative symptoms become prominent only after acute psychosis wanes [7, 8]. With the availability of novel modelling statistics and large longitudinal data, it is now possible to explore such assumptions. Network modelling of psychopathology does not presume a latent variable: individual symptoms are connected by statistical relationships, and large datasets can be modelled to reveal new structures [9–11]. Network analyses completed over different time-points can explore the connectivity, stability, and structure of symptoms over time, which may provide key information for interventions targeted at individuals likely on a pathway to treatment resistance [12].

Network analysis studies in chronic schizophrenia have identified central symptoms such as paranoid ideation, apathy, avolition, and depression, which are reported to activate other symptoms via a contagion effect, leading to the maintenance of psychopathology [13–18]. Others have explored changes in network structure in those with and without remitted status [15, 17, 19], or in response to antipsychotic treatment [4, 14, 20, 21]. Whilst these findings are informative, they are limited to older individuals with enduring illness, and have been conducted with relatively small sample sizes.

Identifying early treatment targets in the ‘critical’ phase of illness has the potential for greatest impact [22]. A small number of studies have applied network modelling to understand psychosis symptoms in young people. Preliminary findings suggest that the strength of network architectures and symptom connectedness may indicate psychosis liability. However, these findings remain exploratory given the small (*N* = 16) cross-sectional nature of Schmidt et al’s study [23], and the second study by Wigman et al was based on a community (rather than a clinical) sample with psychotic-like-experiences [24]. Finally, two recent papers in FEP have demonstrated the potential role of depression and general psychopathology with psychotic symptom expression. In the first, Betz and colleagues (2020) showed that general psychopathology mediated the relationship between the burden of life events and expression of psychotic symptoms, supporting an affective pathway to psychosis [6, 25, 26]. Second, Herniman and colleagues (2021) demonstrated that depression symptoms were highly interrelated with positive and negative symptoms, suggesting that depression symptoms might be better conceptualized as intrinsic to psychosis [27]. Though these studies are informative, they are limited by their cross-sectional design and small samples of young people with FEP.

In this present study, we used a large, diverse, national FEP cohort to explore the structure and inter-relationships between symptoms in early psychosis, using a robust network analyses design to: (a) identify network structures at baseline and 12-months follow-up; and b) identify key central and bridge symptoms that may offer treatment targets for novel interventions.

## Method

### Sample

The EDEN dataset is a longitudinal naturalistic study of 1027 individuals with FEP, recruited from 14 early intervention services (EIS) across England (2005 to 2010; ethical approval REC: 05/Q0102/44.); the methodology and baseline characteristics have been outlined previously [28], but an overview of sample characteristics can be found in Table 1. In summary, observer rated assessments were conducted at baseline (upon entry to EIS), and at a 6 and 12-month timepoint. Complete data on the variables of interest were available for 718 participants by 12-month follow-up.

**Table 1:** EDEN Sample Characteristics at Baseline.

	Baseline (*n* = 1027)
Age of Onset Mean (SD)	21.3 (4.98)
Sex	Female: 318 (31.0)
*n* (%)	Male: 709 (69.0)
Ethnicity	Asian – Bangladeshi: 16 (1.6)
*n* (%)	Asian – Indian: 28 (2.7)
	Asian – Other: 12 (1.2)
	Asian – Pakistani: 101 (9.8)
	Black – African: 23 (2.2)
	Black – Caribbean: 35 (3.4)
	Black – Other: 13 (1.3)
	Mixed – Other: 8 (0.8)
	Mixed – White & Asian: 11 (1.1)
	Mixed – White & Black African: 5 (0.5)
	Mixed – White & Black Caribbean: 19 (1.9)
	Other – Other: 6 (0.6)
	White – British: 723 (70.4)
	White – Irish: 6 (0.6)
	White – Other: 21 (2.0)
Employment	Home maker 22 (2%)
Status	Other 11 (1%)
	Student 199 (19%)
	Unemployed 590 (57%)
	Working (paid) 189 (18%)
	Working (voluntary) 9 (1%)
	n/a or data not known 7 (1%)
Living Status	Alone 130 (13%)
	Data unavailable
	Other 137 (13%)
	With parents/guardian 649 (63%)
	With partner 108 (11%)
	n/a or data not known 3 (0%)
Marital status	Cohabiting 66 (6%)
	Divorced 8 (1%)
	Married and cohabiting 61 (6%)
	Married and separated 21 (2%)
	Single 871 (85%)

The authors assert that all procedures contributing to this work comply with the ethical standards of the relevant national and institutional committees on human experimentation and with the Helsinki Declaration of 1975, as revised in 2008. All procedures involving human patients were approved by Suffolk Local Research Ethics Committee, UK. Approval number: 05/Q0102/44. Written informed consent was obtained from all patients.

### Assessments

*The Positive and Negative Syndrome Scale (PANSS)* [29]. PANSS consists of 30-items measuring severity of positive, negative and general symptoms. Each item is scored between 1 (= absent) to 7 (= extreme). For this study, the Positive Scale (seven items) and Negative Scale (seven items) were used.

*The Calgary Depression Scale for Schizophrenia (CDSS)* [30]. The CDSS includes a total of 9 depressive symptoms (eight structured questions and one interviewer observation) and a scale that ranges from 0 (absent) to 3 (severe).

## Statistical analysis

Descriptive data analysis and network modelling were carried out using R, Version 4.0.3 [31]. (Code for the network analysis is available in the supplementary materials).

### Missing data

For the full EDEN sample (*N* = 1027), there were 895 complete cases at baseline, 757 complete cases at 12 months, and 618 complete cases across the two time points. Item level data were missing for 6.2% of the sample at baseline, and 23·9% at 12-months. Missing data were imputed using an iterative Markov Chain Monte Carlo method, which can concurrently generate Bayesian simulations for binary distributions for cases with incomplete data for all cases [32]. While missing network data can be problematic, there is a lack of simulation studies testing the performance of imputation techniques alongside variable selection methods. We chose EBICglasso over pairwise / likelihood techniques, based on prior research within the structural equation literature showing superior performance of lasso-based methods when the number of variables with missing is large, and when there’s a range of parameters which are also likely to be moderately or highly correlated [33]. Nevertheless, sensitivity analyses were conducted using the complete cases to compare any network differences when using the imputed data (please see results section). Finally, we decided not to include the 6-month network due to the increased complexity it would add to the analysis, reducing the interpretability.

### Network estimation

Two networks were estimated using the full EDEN sample (*N* = 1027), which included complete and imputed cases at baseline and 12-months. We used the ‘bootnet’ package [11] which implements the ‘EBICglasso’ algorithm from the ‘qgraph’ package [34], in turn uses the ‘glasso’ algorithm from the ‘glasso’ package [35]. Network structures were estimated using regularised partial correlation, with coefficients ranging from -1 to 1, representing the association between two nodes after controlling for all other possible information. Partial correlations can be visualised in a weighted network structure with each node representing a variable (e.g. symptom), and each edge showing that two variables are not independent after conditioning all other variables. The edge weights are their partial correlation coefficients. Given the ordinal nature of the data, Spearman’s correlations were used to create covariance matrix via the ‘lavaan’ package [36]. The resulting covariance matrix is inputted into the ‘EBICglasso’ algorithm which uses the least absolute shrinkage and selection operator (lasso) regularisation [37], resulting in sparse networks. ‘EBICglasso’ selects the lasso tuning hyperparameter (λ) which minimises the Extended Bayesian Information Criterion (EBIC) [38]; the EBIC hyperparameter (γ) was set to zero in this study.

There remains contention as to whether regularization estimators, such as glasso, add benefit over more traditional frequentist approaches when estimating psychological networks. Indeed, it has recently been shown that classic methods, such as maximum likelihood estimator (MLE), outperform regularization when applied to low dimensional settings, common in psychology [39, 40]. There is an inflated false positive rate inherent in regularization estimators, such as lasso, when the ratio of parameters to observations is low. We chose a regularization algorithm in this study because of its superiority in performance over non-regularized models when there is a wide range of predictor variables, which are likely to be highly correlated [33, 40]. In such instances, lasso models have much lower Type II error rates, and are less likely to omit truly positive associations, suiting the more exploratory nature of this study [40].

The network structures are plotted using the ‘qgraph’ function (from ‘qgraph’ package) [34]; blue edges indicate positive partial correlations, and red edges indicatingnegative partial correlations. Nodes are placed using a modified version of the Fruchterman-Reingold algorithm [41], constraining the layout to be equal across the networks using the ‘averageLayout’ from the ‘qgraph’ package, enabling comparison. Maximum edge value was set to 0.5367 (the strongest edge identified across both networks); meaning saturation and edge thickness can be compared across graphs.

### Network comparison test

A permutation-based test implemented within the ‘NetworkComparisonTest’ package [42] compared the baseline and 12 month symptom networks on global structure, overall connectivity level by average strength of all edge weights, and the difference in strength of individual edge weights. Finally, centrality estimates were computed using the “test centrality” command, which statistically assesses the centrality of symptoms across the two networks.

### Centrality and bridge centrality estimation

The strength of nodes within each network were established by summing the absolute edge weights connected to a particular node [43–45]. The importance of each node in acting as a bridge to the three communities of symptoms (other than the community it originates), was calculated using the recently defined concept of bridge centrality implemented in the ‘networktools’ package [46]. We used the bridge strength estimate which indicates a node’s total connectivity with other communities. The top 20% scoring nodes (a cut-off giving an acceptable balance between sensitivity and specificity) on bridge strength was also indicated graphically [47].

### Network accuracy and stability assessment

Bootstrapping methods were performed using the ‘bootnet’ package [48] to assess the accuracy and stability of the derived network parameters. We bootstrapped 95% CIs around the edge weights, the significance (α = 0·05) between the edges, and the significance (α = 0.05) between the centrality metric of the nodes for each network. The stability of the centrality indices was assessed via a case-dropping bootstrap, which were summarised using CS-coefficients (correlation stability), quantifying the proportion of the data that can be dropped to retain a correlation of at least 0.7 with 95% certainty. The CS-coefficient should be ideally above 0.5 but at least above 0.25 [49].

## Results

### Sample

Full demographic characteristics of the EDEN sample have been outlined previously [28]. In summary, the sample (*n* = 1027) had a mean age of 21.3 years, 69% were male, and 70% were White British. The baseline network had greater positive, negative, and depressive symptoms compared to the 12-month network (Table 2).

**Table 2:** Comparison of symptom scores across the baseline and 12-month networks.

	Baseline	12 months	Paired *t*-test
PANSS Positive Symptoms Total			
Mean (SD)	15.3 (6.0)	11.2 (4.4)	*t* (1026) = 19.9
			*p* = <0.001
PANSS Negative Symptoms Total			
Mean (SD)	14.9 (6.5)	11.9 (5.2)	*t* (1026) = 14.5
			*p* = <0.001
CDSS Total			
Mean (SD)	6.2 (5.3)	3.4 (4.2)	*t* (1026) = 16.5
			*p* = <0.001

*PANSS* Positive and Negative Syndrome Scale, *CDSS* Calgary Depression Scale for Schizophrenia.

### Baseline network

**Fig. 1:**
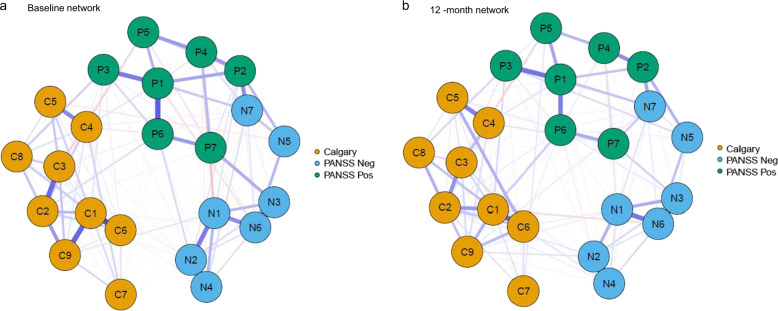
Symptom network maps across timepoints. A network structure for baseline is depicted in 1(a), and 1(b) for the 12 month network. Nodes (circles) represent individual symptoms. Orange nodes represent depressive items from the Calgary Depression Scale for Schizophrenia (CDSS). Blue nodes represent 7 negative symptoms from the PANSS scale, and green nodes represent items from the PANSS positive scale. Edge weights (lines) represent the strength of association between symptoms. Blue edges represent positive associations and red edges represent negative associations; denser lines represent stronger connections. P1_ = Delusions; P2 = Conceptual Organization; P3 = Hallucinatory Behaviour; P4 = Excitement; P5 = Grandiosity; P6 = Suspiciousness/Persecution; P7 = Hostility; N1 = Blunted Affect; N2 = Emotional Withdrawal; N3 = Poor Rapport; N4 = Passive/Apathetic Social Withdrawal; N5 = Difficulty in Abstract Thinking; N6 = Lack of Spontaneity and Flow of Conversation; N7 = Stereotyped Thinking; C1 = Depression; C2 = Hopelessness; C3 = Self Depreciation; C4 = Guilt Ideas of Reference; C5 = Pathological Guilt; C6 = Morning Depression; C7 = Early Awakening; C8 = Suicide; C9 = Observed Depression.

At baseline, 52.2% of all possible edges were retained in the regularized networks. The network structure can be visualised in Fig. 1a. Distinct symptom communities can be visualised based on the three original symptom groups: PANSS Positive, PANSS Negative and CDSS.

**Fig. 2:**
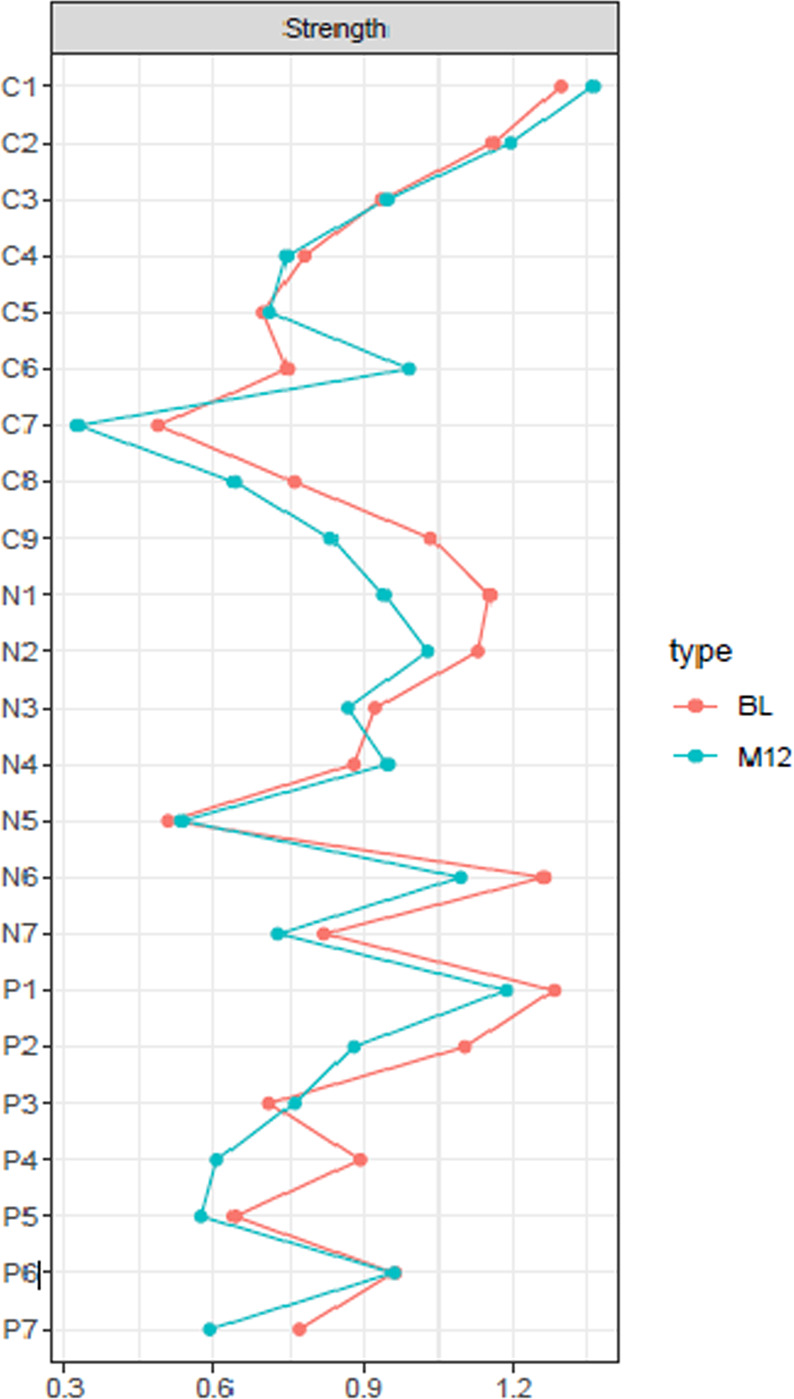
Node Strength centrality estimates for the baseline and 12-month networks. Red lines = baseline network; blue lines = 12-month network. Standardized z-scores are plotted for ease of interpretation. Higher scores represent higher centrality estimates (i.e. the symptom has greater influence in the network).

**Fig. 3:**
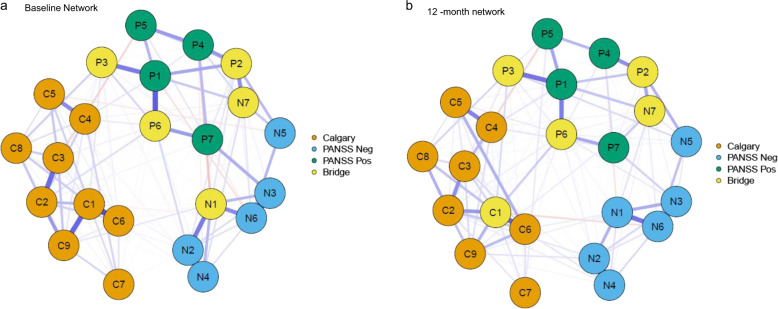
Top scoring bridge nodes across the networks. Network structures for baseline (3**a**), and 12-months (3**b**), display the top 20% scoring nodes on bridge strength (a cut-off recommended as giving an acceptable balance between sensitivity and specificity). Yellow nodes represent the bridge nodes. Orange nodes represent depressive items from the CDSS scale. Blue nodes represent 7 negative symptoms from the PANSS scale, and green nodes represent items from the PANSS positive scale. See Fig. 1 caption for node key.

Depression (C1), Delusions (P1), and Lack of Spontaneity (N6) had the highest node strength centrality in the baseline network (Fig. 2). The top 20% scoring nodes on bridge strength (Fig. 3) were: blunted affect (N1), stereotyped thinking (N7), conceptual disorganization (P2), hallucinatory behaviour (P3), and suspiciousness (P6). Negative symptoms formed bridges with positive symptoms: stereotyped thinking bridged with conceptual disorganisation, and blunted affect was negatively associated with hostility. Depressive symptoms formed bridges with positive symptoms: Hallucinations and suicide, and suspiciousness and hopelessness were positively associated (Fig. 3a).

### 12-month network

At 12-months, 50.02% of all possible edges were retained in the regularized network. Similar visualisations for baseline network can also be identified in the 12-month network, with strong positive associations between items as visualised in the baseline network (Fig. 1b). Depression (C1) had the highest node strength in the 12-month network (Fig. 2). The top 20% bridge symptoms included: depression (C1), conceptual organisation (P2), stereotyped thinking (N7), hallucinatory behaviour (P3), and suspiciousness (P6). Similar to the baseline network, stereotyped thinking bridged with conceptual disorganisation, and the depressive items bridged with positive symptoms. Hallucinations and the suicide item were positively related, in addition to positive associations between observed depression with suspiciousness and hallucinations (Fig. 3b).

### Network comparison

Our results indicate that the baseline and 12-month networks differed significantly in overall structure (M = 0.16405, *p* = <0.0001) and connectivity (S = 0.74536, *p* = 0.02), but did not differ significantly in overall strength centrality and bridge centrality (C = 0.096153; *p* = 0.22297).

The global strength and overall connectivity of the baseline network was stronger. Similarly, for the structure, the baseline network retained more edges than the 12-month network. Ten edges were significantly different across the baseline and 12-month networks. Excitement (P4) with emotional withdrawal (N2), delusions (P1) with lack of spontaneity (N6), hallucinatory behaviour (P3) with stereotyped thinking (N7), suspiciousness (P6) with depression (C1), excitement (P4) with guilt ideas of reference (C4), passive social withdrawal (N4) with pathological guilt (C5), pathological guilt (C5) with morning depression (C6), grandiosity (P5) with early awakening (C7), abstract thinking (N5) with suicide (C8), and depression (C1) with observed depression (C9).

### Network accuracy and stability

The bootstrap analyses showed that the networks were very stable and edge weights were accurately estimated. The results for the edge weight bootstrap, edge weights significance testing, and strength centrality difference testing can be visualised in the supplementary material (Please see Fig. 1–6 in the supplementary material). For the subset bootstrap, the two networks showed acceptable centrality stability (Figures 7 and 8 in the supplementary materials). These results are consistent with the CS-coefficient, which was 0.75 for strength for the baseline network, and 0.75 for strength in the 12-month network, suggesting that the networks remained stable.

### Sensitivity analyses

Because of the high level of missing data, sensitivity analyses were conducted using the complete cases (*N* = 718). The data are available in the supplementary material (Figures 9–19), but in sum, the baseline and 12-month networks remained dense (47% and 50.6%, respectively), and stable (0.75). Key central and bridge symptoms remained the same, though unlike the networks with imputed cases, the overall network structure and connectivity was not significantly different across the two time points.

## Discussion

This study presents the first analysis of symptom networks in a large, representative FEP sample, over two timepoints. Key findings were as follows: the networks differed significantly in terms of overall structure and connectedness, but central symptoms did not differ significantly. Depression featured consistently as a central symptom in the baseline and 12-month network. Conceptual disorganisation, stereotyped thinking, along with hallucinations and suspiciousness, remained key bridge symptoms across the networks.

It has previously been shown that network structures and connectedness differ for those in remission [15, 17, 19]. Within the present study, the difference in network structure and connectedness may reflect an improvement in symptoms by 12 months. However, interestingly, we showed that symptom centrality remained unchanged across the networks. Identifying key symptoms which become prominent in the networks over this critical illness period may serve as fruitful treatment targets to promote recovery.

### Influential network symptoms

The emergence of depression as an influential symptom in the baseline and 12 month network is in line with an affective pathway to psychosis, where psychosis symptoms may result from heightened emotional reactivity [7, 26, 50]; potentially reflecting a continuation of a longstanding developmental trajectory [51]. Recent network studies add to this evidence, demonstrating that FEP is rooted in the context of both earlier sub-threshold symptoms as well as non-psychotic symptoms [51]. A recent cross sectional network study by Bet and colleagues also demonstrated that the relationship between adverse life events and psychosis symptoms was only present via general symptoms such as depression and guilt feelings [25]. Burden of recent life events predicted depression and guilt 3 months later, demonstrating the temporal relationship between life event burden and severity and persistence of affective psychopathology [25]. In the present longitudinal paper, the presence of central depressive symptoms early in the illness course may suggest that affective symptoms may underly the expression of other psychotic symptoms [52]. Though psychotic symptoms had significantly reduced by follow-up, the centrality of affective symptoms might mean that the network is descended into a state of vulnerability, where a potential worsening of depressive symptoms (e.g., triggered by a burdensome life event), may lead to a global worsening of psychosis symptoms.

An alternative explanation is that depressive symptoms may be secondary to the resolution of frank psychosis. Post-psychotic depression is prevalent following the initial episode and might account for the high rates of suicidality following FEP [8, 53]. It often arises as a result of negative illness appraisals once insight is regained [8]. However, within our networks, depression remained a central symptom from baseline, and consistent bridge-symptoms between depression and psychosis symptoms were observed.

When bridge-symptoms are present, the likelihood of other communities of activated symptoms increases. Bridge-symptoms can help to explain the continuity and comorbidity of depression and psychosis [10]. Within this study, a bridge between hallucinations and suicide was consistent across the two time points. This complex interplay has been demonstrated previously; predominantly, suicidality is associated with auditory hallucinations [54]. We have shown that individuals with FEP and depression experience greater perceived malevolence in voices and greater engagement [8], and command hallucinations, where a voice is perceived as power and omnipotent, is associated with suicidal behaviour [55].

Within our networks, bridges also emerged between suspiciousness with depression and hopelessness; again, a phenomenon reported in prior research where those with

persecutory delusions encounter depression, especially when they feel less powerful than their persecutors [8, 56]. This relationship is also replicated in previous network studies in males with schizophrenia, and recently in young people with FEP [15, 27]. Here, these studies also showed depression as being intrinsic to psychosis symptoms. A longitudinal network approach comparing individuals across different illness stages would be necessary to establish the causal relationship between depression and the expression of psychosis symptoms.

Finally, it is also notable that conceptual disorganisation and stereotyped thinking, featured consistently as central and bridge symptoms in the networks. Formal thought disorder, which is characterised by disturbances in thought, language and communication [57], has been identified as a core feature in those with enduring illness and is linked to adverse outcomes, including: higher relapse and re-hospitalisation rates [58], and poorer social and occupational functioning [59, 60]. Although the impact of thought disorder on illness manifestation in the early stages of illness is generally under-recognised, in a study of individuals at-risk of psychosis, those with disturbances in thought and communication were more likely to transition to psychosis and have poorer functional outcomes [61]. This may suggest that the emergence of thought and communication disturbances in early psychosis may be a marker of long-term poor outcomes [59]. Indeed, it has previously been proposed that thought disorder is a manifestation of a core deficit of ‘classical’ schizophrenia, characterised by pervasive brain changes, cognitive impairment, and entrenchment of poor functioning [62, 63]. This psychopathological trajectory also invokes the idea of Hebephrenia, and the presence of these characteristic may indicate a more pervasive course of illness [64].

### Implications

Identifying key central and bridge symptoms in developing psychopathology is potentially important, as they may activate other symptoms in the network, creating self-reinforcing feedback loops [15]. Targeting interventions at key symptoms may break down this maintenance cycle and provide a boost in momentum required for global improvement [4]. Symptoms of formal thought disorder (e.g. conceptual disorganisation, stereotyped thinking and excitement), in addition to depressive symptoms, showed prominence in the networks at baseline and follow-up; these symptoms may offer more refined targets for novel stratified treatments in early phases of psychosis. It is apparent that a number of individuals in FEP continue to have poor outcomes and remain unresponsive to ‘gold standard treatments’ [65]. Those with comorbid depression in psychosis are shown to have poor outcomes [66, 67]. Conventional interventions for those with particularly complex symptom presentations, such as those presenting with early thought disorder, are also shown to be less effective [68]. This highlights the need for better recognition of these symptoms in early psychosis, in addition to improved, and stratified interventions for subgroups who are unlikely to respond to conventional treatments. Thus, a better understanding of the mechanisms by which these underrecognized early symptoms might facilitate change in the entire symptom network may prove beneficial [4]. Future work may seek to clarify whether network structures differ over time in those with and without a remitted status. This would provide clarity on the causality of network structures over time, and how this may relate to illness outcome and progression. Further research is also necessary to establish whether the influence of depression on psychosis symptoms is an integral feature of the illness, or whether it most prominent at particular time points, such as at 12 months, as we demonstrated within our networks.

### Strengths and limitations

A strength of this study is the examination of a heterogenous sample from a large longitudinal national cohort of young people early in their illness course. In addition, we add to previous network studies in psychosis by including data from male and female participants at two time-points, and use novel statistics to compare network strength, centrality, and connectivity. Although we report on a large national sample of young people with psychosis, data were missing, and subsequently imputed for 23% of our sample. However, results of our sensitivity analyses showed consistent findings regarding network density, stability, and symptom centrality. A second limitation is that we did not include socio-demographic factors within our networks. Such factors (e.g. sex, age of onset, level of education) are shown to influence illness outcomes in schizophrenia, however, their influence on symptom expression remains inconclusive, particularly in the early phase of illness [69]. Two studies within FEP showed that despite differences between males and females on age of onset, premorbid functioning, and duration of untreated psychosis, there were no differences in symptoms severity at presentation [70, 71]. Whilst this warrants investigation at a network level, inclusion of these factors within our network is beyond the focus of our research question. Third, we acknowledge that the group-level nature of our analysis does not allow for conclusions to be drawn on an individual level. And finally, though PANSS scores differed between the networks, overall, the scores are relatively low. Comparisons with network structures in those with more severe and enduring psychopathology, as well as those at clinical high risk of psychosis, may be more informative in understanding illness stage and progression. Within the current design, we were not able to establish whether depression emerges as prominent not just at 12 months, but potentially earlier, during the prodrome or sub-threshold stages.

## Conclusion

We provide novel findings of symptom networks in early psychosis with robust data from a large longitudinal sample. Depressive symptoms, in addition to conceptual disorganisation and stereotyped thinking, which are often under-recognised in early psychosis, may potentially serve as novel symptom targets, which if adequately addressed, may have the potential to lead to global symptom improvements and better recovery.

## Supplementary information

Figure 1. Baseline bootstrapped CI of edge weights

Figure 2. Baseline bootstrapped difference tests for non zero edges

Figure 3. Baseline bootstrapped difference test between node strength

Figure 4. Twelve month bootstrapped CI of edge weights

Figure 5. Twelve month bootstrapped difference tests for non zero edges

Figure 6. Twelve month bootstrapped difference test between node strength

Figure 7. Baseline average correlations with sample case dropping bootstrapped

Figure 8. Twelve month average correlations with sample case dropping bootstrapped

Figure 9. Network visualisations for the sensitivity analysis

Figure 10. Strength centrality estimates for the sensitivity analysis

Figure 11. Top 20% scoring bridge nodes for the sensitivity analysis

Figure 12. Baselinebootstrapped CI of edge weights for the sensitivity analysis

Figure 13. Baseline bootstrapped difference tests for non zero edges for the sensitivity analysis

Figure 14. Baseline bootstrapped difference test between node strength for the sensitivity analysis

Figure 15. Baseline average correlations with sample case dropping bootstrap for the sensitivity analysis

Figure 16. Twelve month bootstrapped confidence intervals for the edge weights for the sensitivity analysis.

Figure 17. Twelve month Bootstrapped difference tests for non zero edges for the sensitivity analysis.

Figure 18. Twelve month bootstrapped difference test between node strength for the sensitivity analysis

Figure 19. Twelve month average correlations with sample case dropping bootstrap for the sensitivity analysis

Code Final R
