# Supplementary material for: Structure and stability of symptoms in first episode psychosis: a longitudinal network approach
Source: Transl Psychiatry. 2021 Nov 6;11:567. doi: 10.1038/s41398-021-01687-y (PMC8572227; doi:10.1038/s41398-021-01687-y)
Supplement: Supplementary file 1 — Figure 1. Baseline bootstrapped CI of edge weights [file 41398_2021_1687_MOESM1_ESM.pdf]

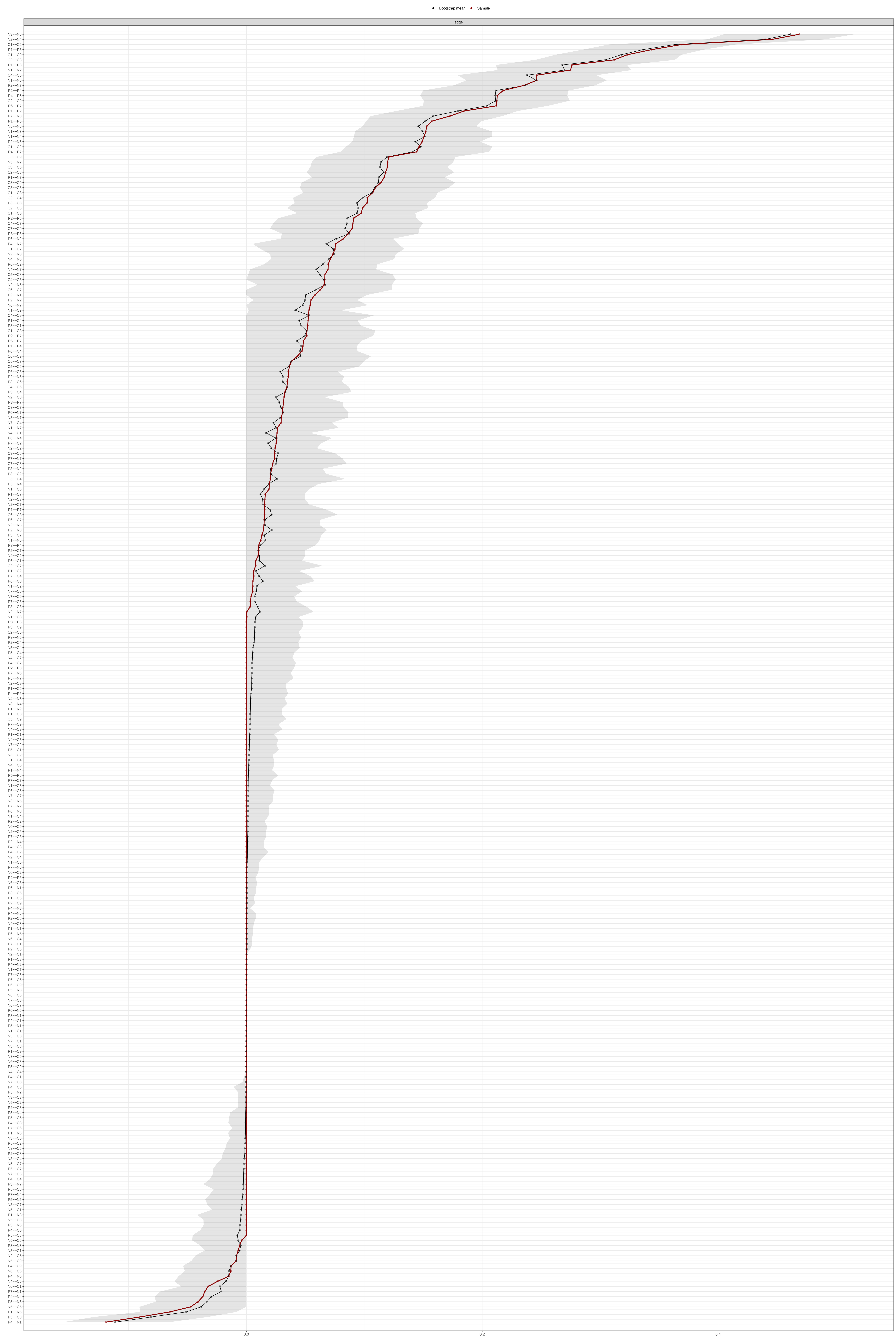

Bootstrap mean
Sample
edge
0.0
0.2
0.4
