# Supplementary figures and images for: Structure and stability of symptoms in first episode psychosis: a longitudinal network approach
Source: Transl Psychiatry. 2021 Nov 6;11:567. doi: 10.1038/s41398-021-01687-y (PMC8572227; doi:10.1038/s41398-021-01687-y)

• Bootstrap mean • Sample

edge

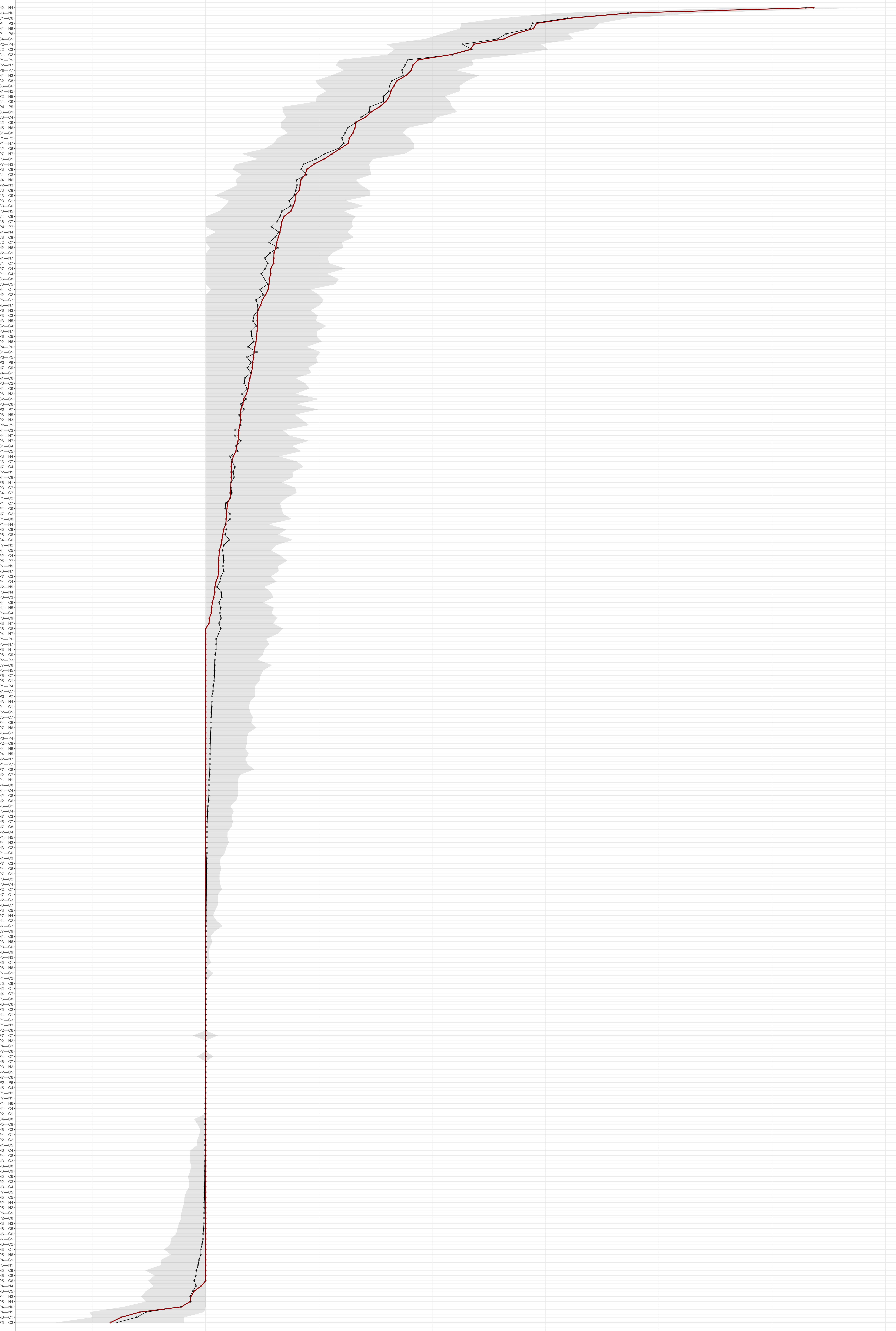

Supplement: Supplementary file 4 — Figure 4. Twelve month bootstrapped CI of edge weights [file 41398_2021_1687_MOESM4_ESM.pdf]

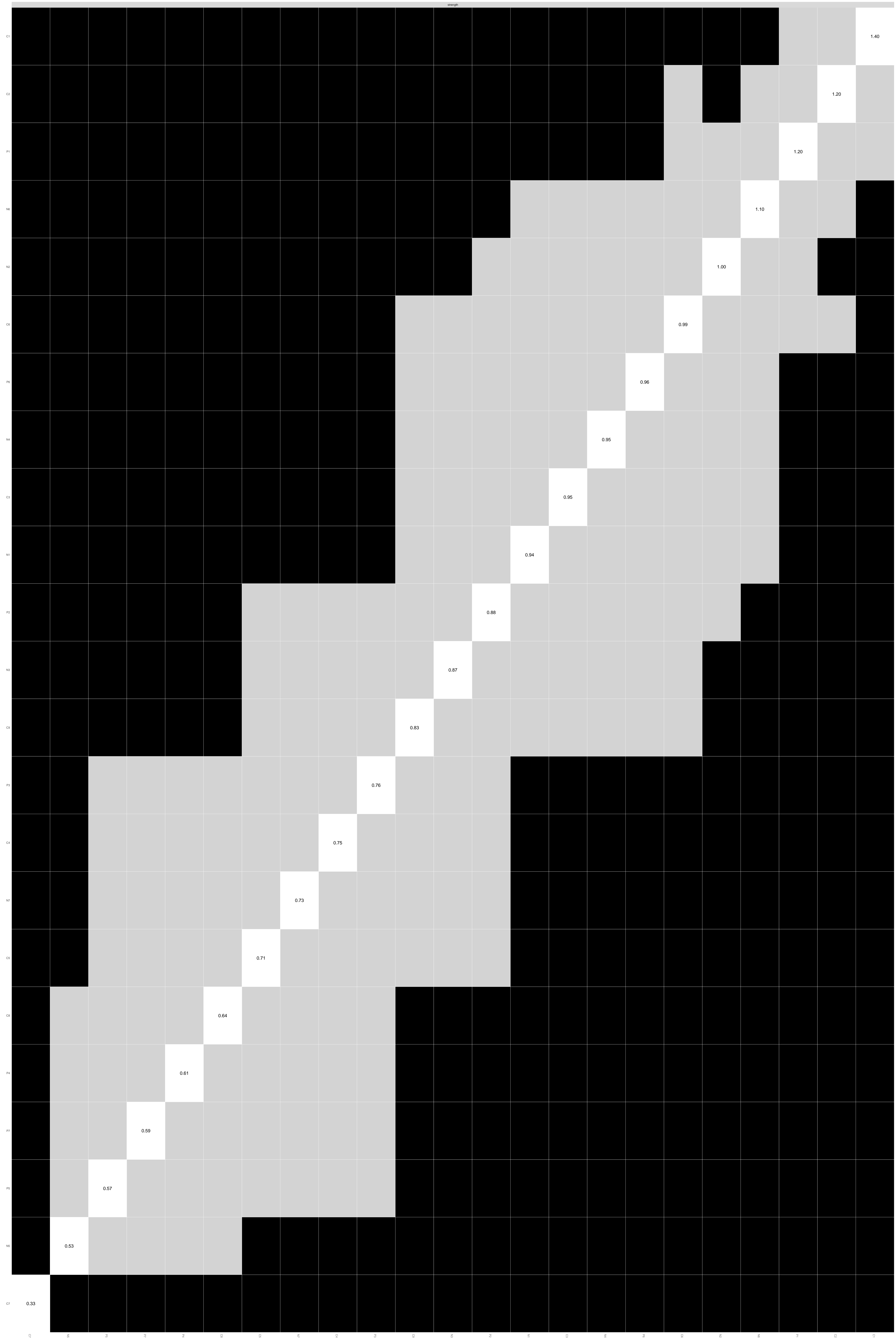

Supplement: Supplementary file 6 — Figure 6. Twelve month bootstrapped difference test between node strength [file 41398_2021_1687_MOESM6_ESM.pdf]

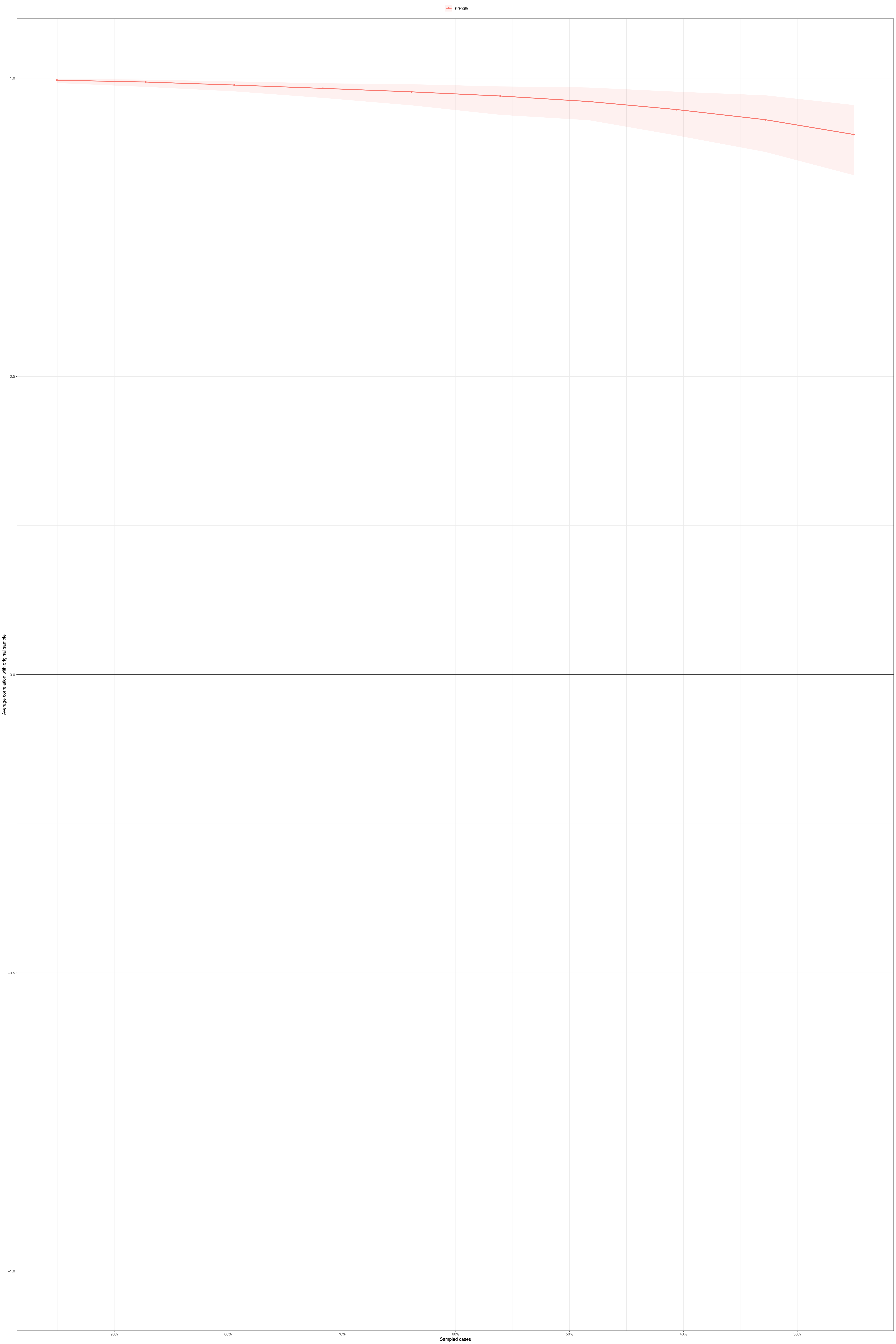

Supplement: Supplementary file 7 — Figure 7. Baseline average correlations with sample case dropping bootstrapped [file 41398_2021_1687_MOESM7_ESM.pdf]

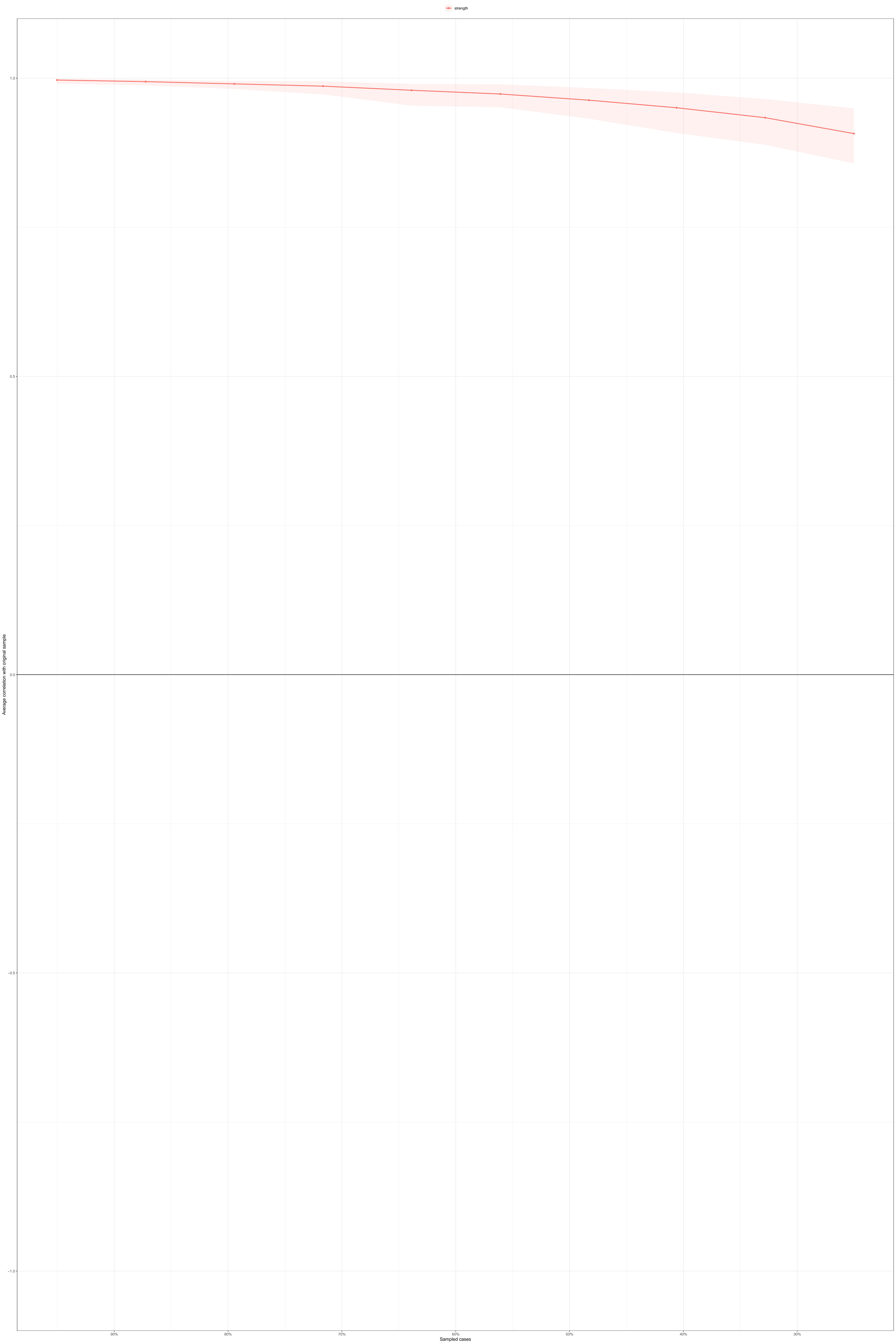

Supplement: Supplementary file 8 — Figure 8. Twelve month average correlations with sample case dropping bootstrapped [file 41398_2021_1687_MOESM8_ESM.pdf]

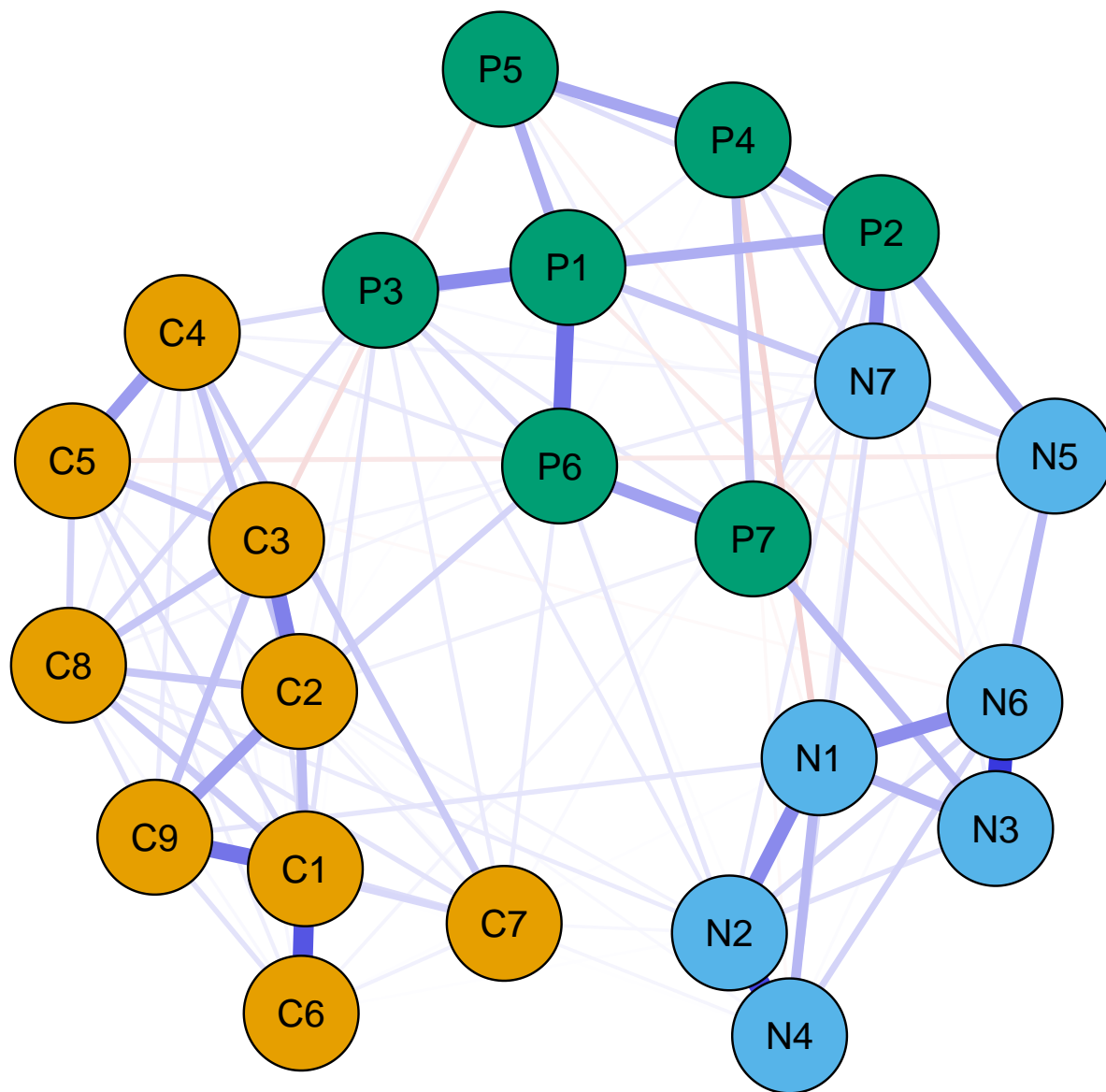

- Calgary
- PANSS Neg
- PANSS Pos

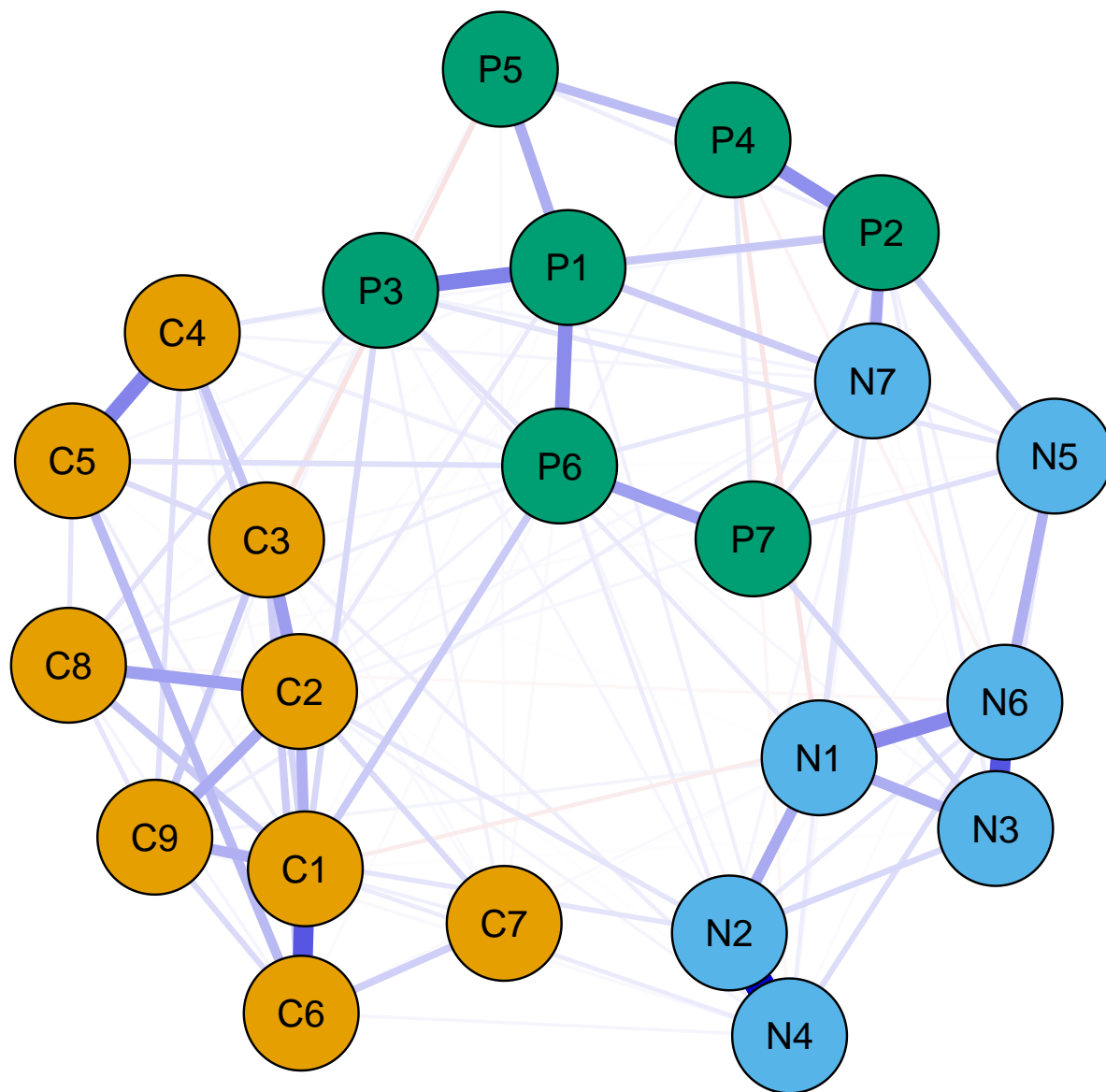

- Calgary
- PANSS Neg
- PANSS Pos

Supplement: Supplementary file 9 — Figure 9. Network visualisations for the sensitivity analysis [file 41398_2021_1687_MOESM9_ESM.pdf]

## Strength

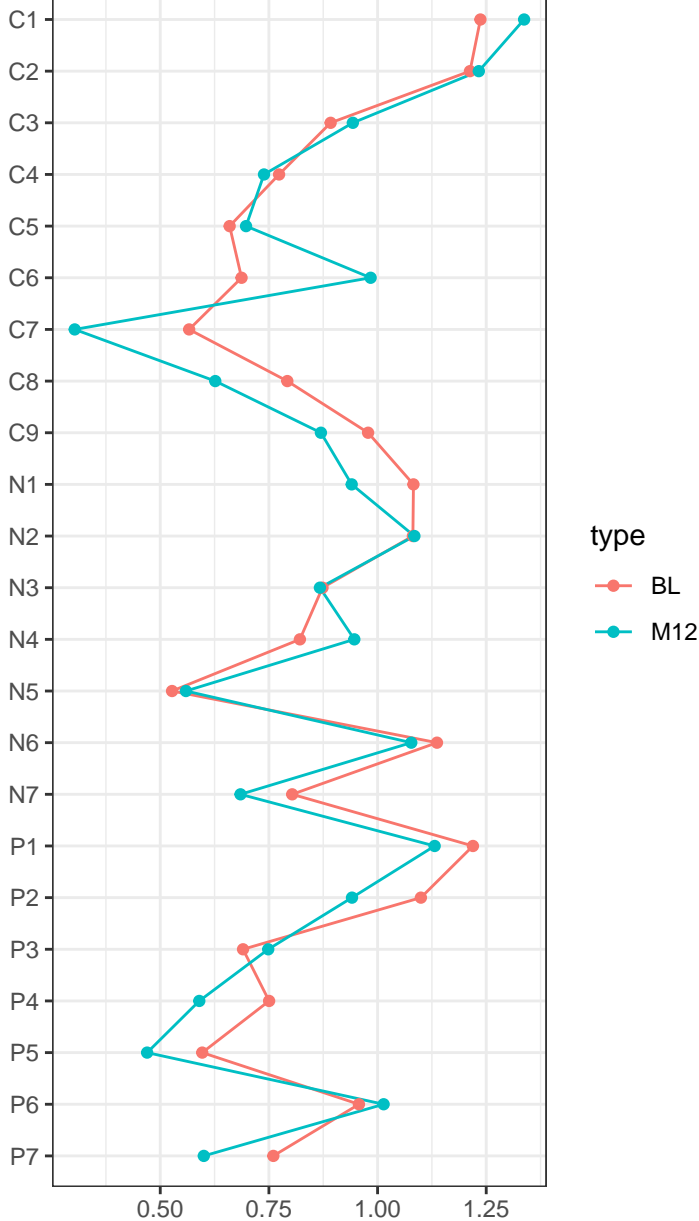

Supplement: Supplementary file 10 — Figure 10. Strength centrality estimates for the sensitivity analysis [file 41398_2021_1687_MOESM10_ESM.pdf]

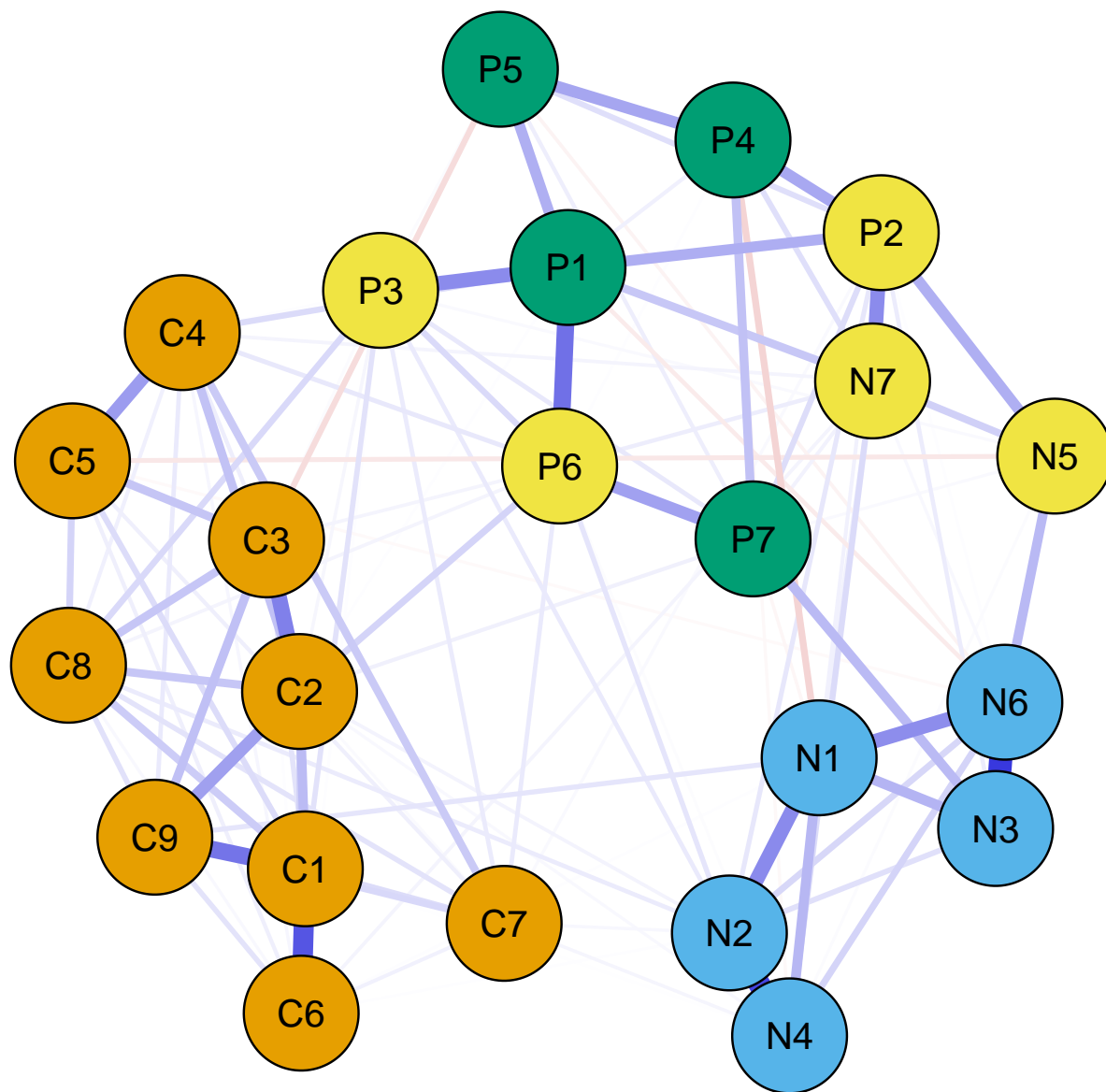

- Calgary
- PANSS Neg
- PANSS Pos
- Bridge

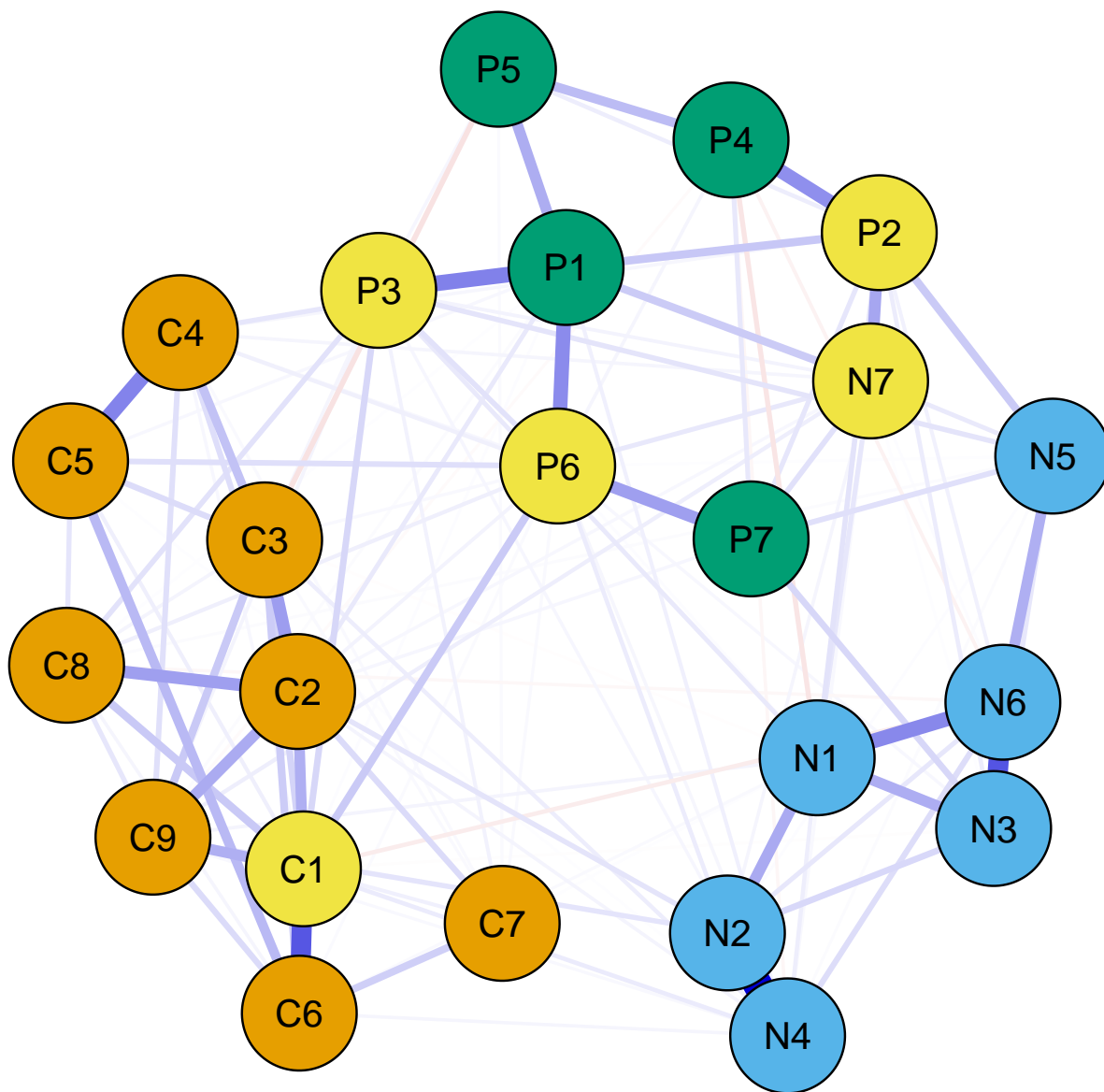

- Calgary
- PANSS Neg
- PANSS Pos
- Bridge

Supplement: Supplementary file 11 — Figure 11. Top 20% scoring bridge nodes for the sensitivity analysis [file 41398_2021_1687_MOESM11_ESM.pdf]

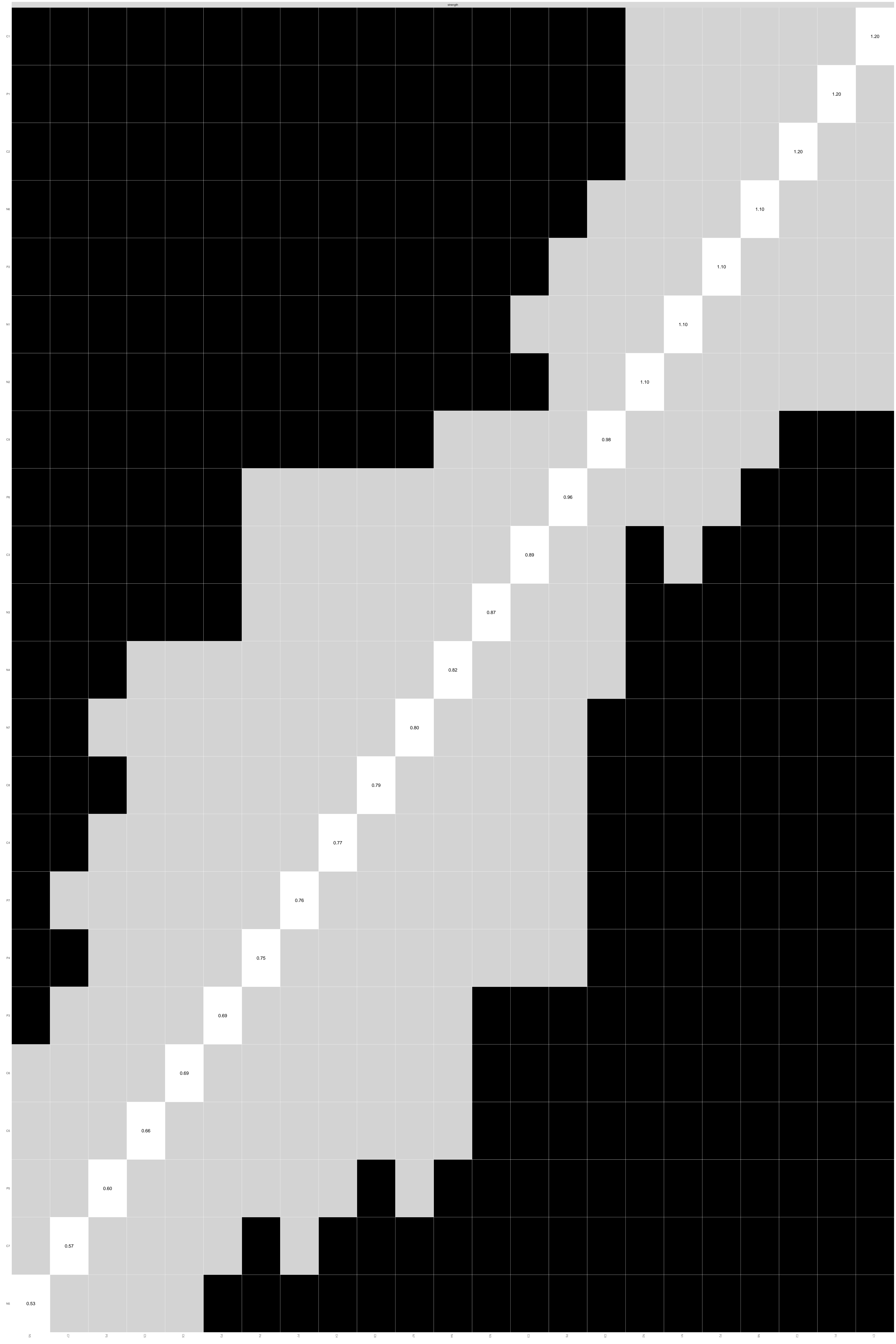

Supplement: Supplementary file 14 — Figure 14. Baseline bootstrapped difference test between node strength for the sensitivity analysis [file 41398_2021_1687_MOESM14_ESM.pdf]

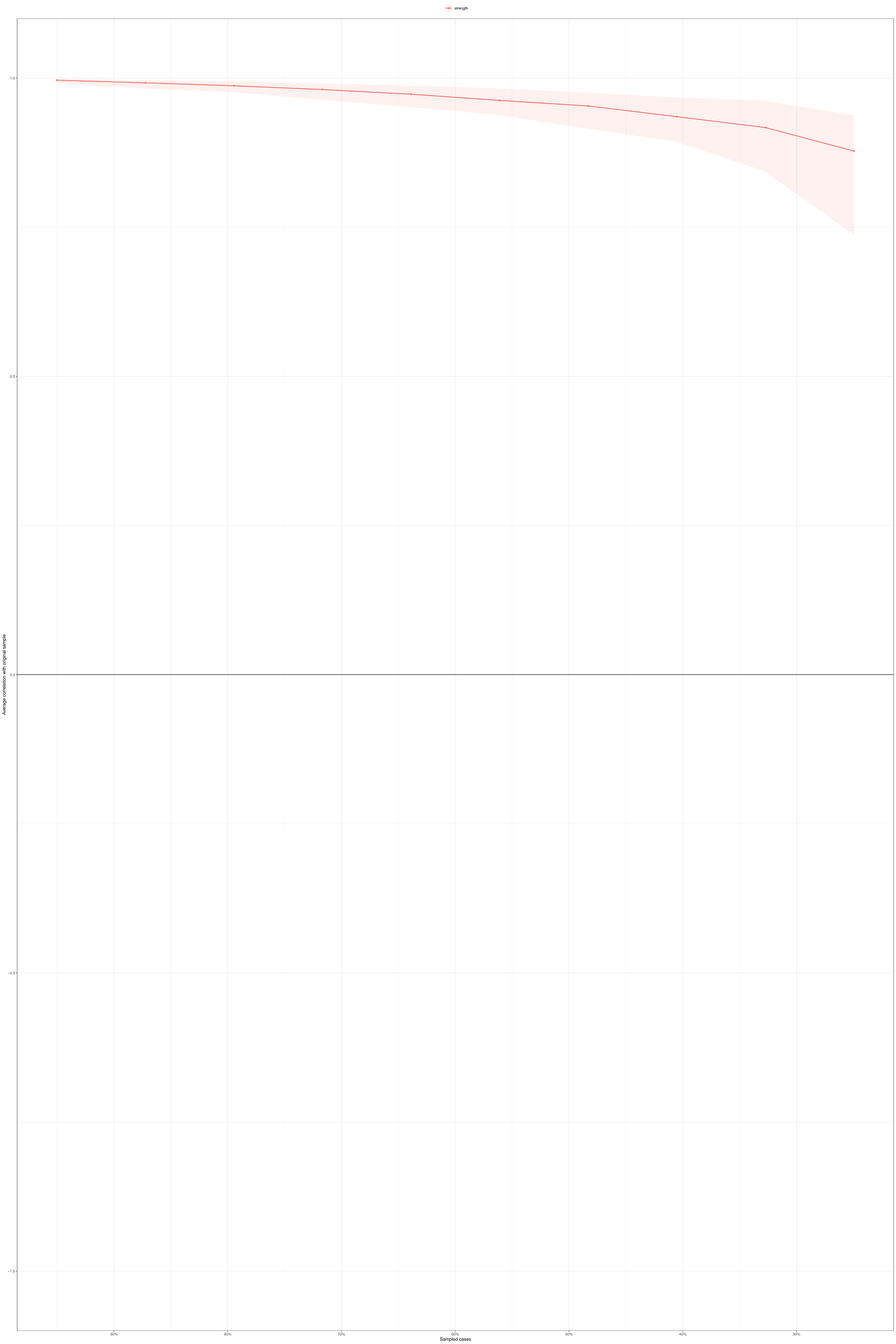

Supplement: Supplementary file 15 — Figure 15. Baseline average correlations with sample case dropping bootstrap for the sensitivity analysis [file 41398_2021_1687_MOESM15_ESM.pdf]

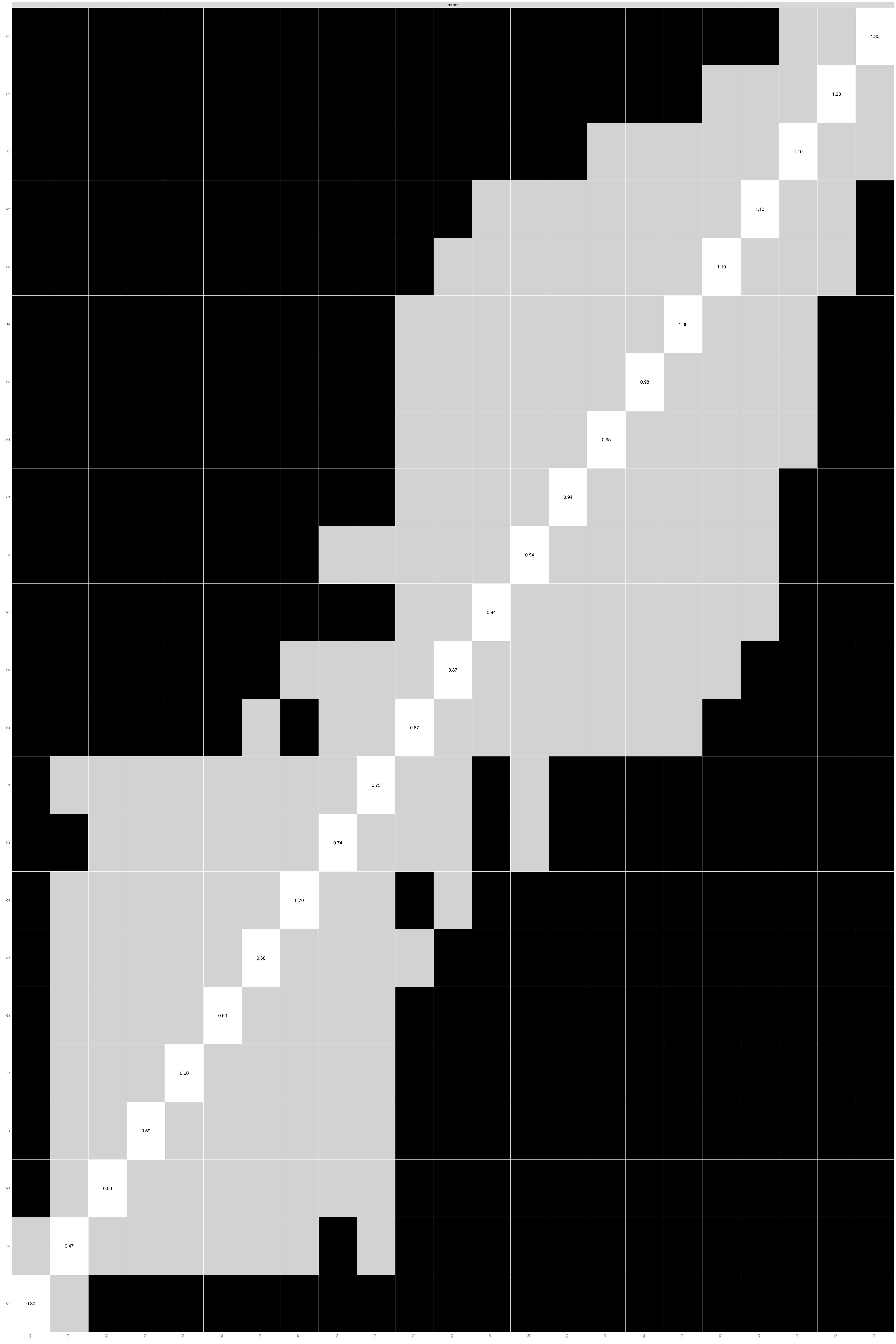

Supplement: Supplementary file 18 — Figure 18. Twelve month bootstrapped difference test between node strength for the sensitivity analysis [file 41398_2021_1687_MOESM18_ESM.pdf]

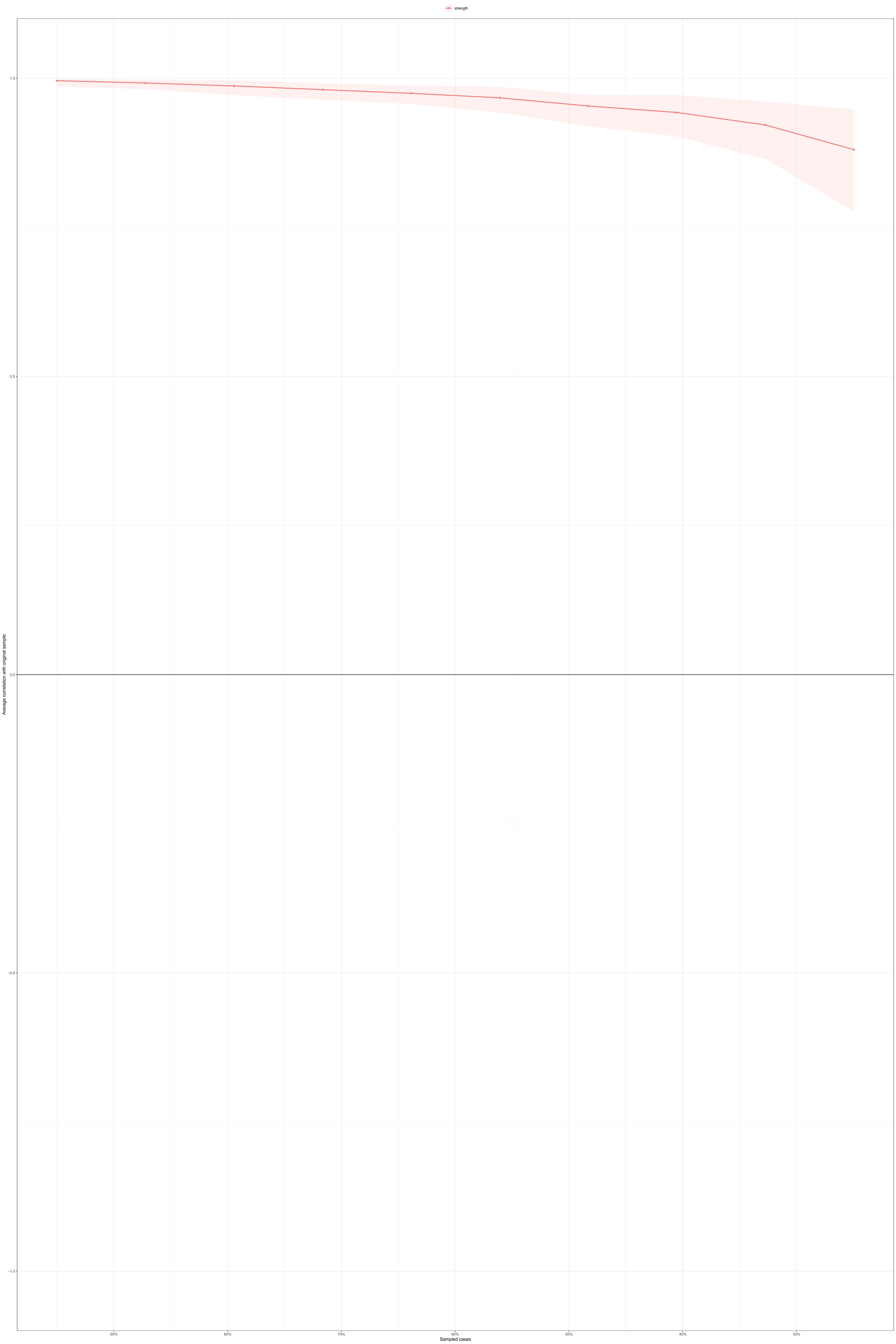

Supplement: Supplementary file 19 — Figure 19. Twelve month average correlations with sample case dropping bootstrap for the sensitivity analysis [file 41398_2021_1687_MOESM19_ESM.pdf]
