# Supplementary material for: Structure and stability of symptoms in first episode psychosis: a longitudinal network approach
Source: Transl Psychiatry. 2021 Nov 6;11:567. doi: 10.1038/s41398-021-01687-y (PMC8572227; doi:10.1038/s41398-021-01687-y)
Supplement: Supplementary file 16 — Figure 16. Twelve month bootstrapped confidence intervals for the edge weights for the sensitivity analysis. [file 41398_2021_1687_MOESM16_ESM.pdf]

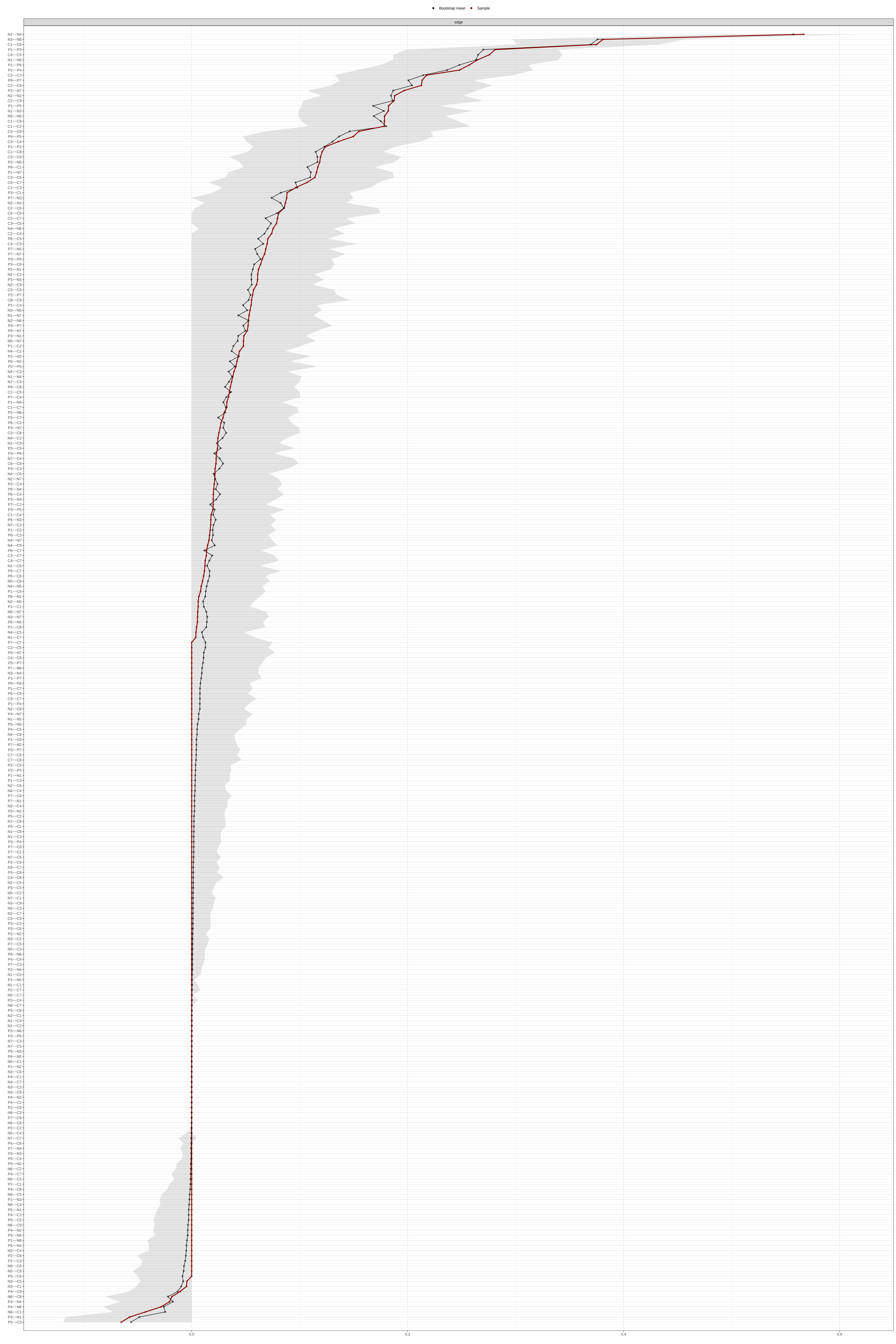
Bootstrap mean
Sample
edge
N2---N4
N3---N6
C1---C6
P1---P3
C4---C5
N1---N6
P1---P6
P2---P4
C2---C3
P6---P7
C2---C8
P2---N7
N1---N2
C2---C9
P1---P5
N1---N3
N5---N6
C1---C9
C1---C2
C5---C6
P4---P5
C3---C4
P1---P2
C1---C8
C3---C9
P2---N5
P6---C1
P1---N7
C3---C6
C6---C7
C1---C3
P3---C1
P7---N3
N2---N3
C2---C6
C6---C9
C2---C7
C3---C5
N4---N6
C2---C4
P6---C5
C4---C9
P7---N5
P7---N7
P3---P6
P3---C8
P2---N1
N2---C2
P3---N5
N2---C9
C5---C8
P2---P7
C8---C9
P1---C4
N3---N5
N1---N7
N2---N6
P4---P7
P6---N7
P3---N1
N5---N7
P1---C2
N4---C1
P2---N3
P6---N2
P2---P5
N4---C3
N1---N4
N7---C9
P6---C8
C1---C5
P7---C4
P1---N4
C1---C7
P2---N6
P3---C7
P6---C2
P3---N7
C3---C8
N4---C2
N1---C9
P3---C9
P4---P6
N7---C4
C6---C8
P3---C3
N4---C6
N2---N7
P2---C4
P6---N4
P6---C4
P3---N4
P7---C2
P3---P5
C1---C4
P6---N3
N7---C2
P1---C5
P6---C3
N4---N7
N4---C9
P6---C7
C3---C7
C4---C7
N1---C6
P5---C7
P6---C6
N5---C8
N4---N5
P1---C9
P6---N1
N2---N5
P1---C1
N6---N7
N3---N7
P6---N5
P1---C8
N4---C5
N1---C7
P7---C7
C2---C5
P5---N7
C4---C6
P5---P7
P7---N6
N3---N4
P1---P7
P5---P6
P1---C7
P6---C9
C5---C7
P1---P4
N2---C8
P4---N7
N1---N5
P5---N5
P4---C5
N4---C8
P1---C6
P7---N2
P3---P7
C7---C9
C7---C8
P2---C5
P2---P3
P1---C3
P1---N1
N2---C6
N4---C4
P7---C8
P7---N1
N2---C4
P3---N2
P5---C2
N7---C8
P5---C1
N1---C8
N1---C3
P3---P4
P7---C6
P7---C1
N7---C6
P2---C9
N3---C7
P5---C8
C4---C8
N2---C5
P3---C5
N5---C2
N7---C1
N3---C9
N2---C3
N2---C7
C5---C9
P3---C2
P3---C6
P2---N2
N3---C2
P7---C5
N5---C3
P6---N6
P4---C4
P7---C3
P2---N4
N1---C5
P1---N5
N1---C1
P2---C7
N5---C7
P3---C4
N6---C7
P5---C9
N2---C1
N1---C4
N1---C2
P3---N6
P2---P6
N7---C3
N7---C5
P5---N3
P4---N5
N5---C1
P1---N2
N3---C6
P4---C1
N4---C7
N3---C3
N3---C8
P4---N3
P4---C2
P2---C6
N6---C3
P7---C9
N5---C6
P2---C2
N5---C4
N7---C7
P4---C6
P7---N4
P3---N3
P5---C4
P5---N2
N6---C2
P4---C7
N5---C5
P2---C1
P4---C8
N6---C5
P1---N3
N6---C4
P5---N1
P4---C3
P5---C5
N6---C9
P4---N2
P5---N6
P1---N6
P5---N4
N3---C4
P2---C8
P2---C3
N6---C6
N5---C9
P5---C6
N3---C5
N3---C1
P4---C9
N6---C8
P4---N4
P4---N6
N6---C1
P4---N1
P5---C3
0.0
0.2
0.4
0.6
