# Supplementary material for: Structure and stability of symptoms in first episode psychosis: a longitudinal network approach
Source: Transl Psychiatry. 2021 Nov 6;11:567. doi: 10.1038/s41398-021-01687-y (PMC8572227; doi:10.1038/s41398-021-01687-y)

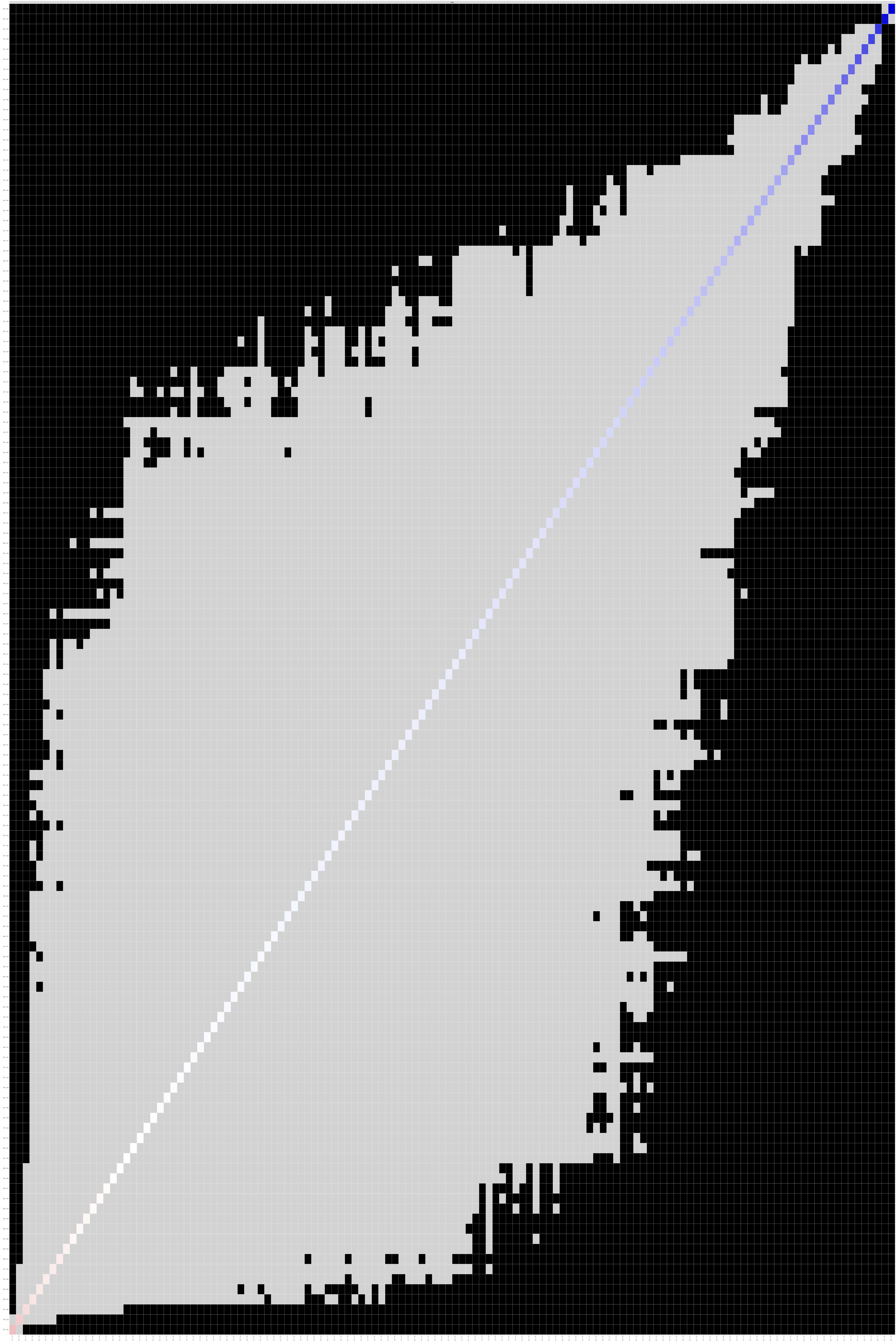

Supplement: Supplementary file 2 — Figure 2. Baseline bootstrapped difference tests for non zero edges [file 41398_2021_1687_MOESM2_ESM.pdf]
