# Supplementary material for: Structure and stability of symptoms in first episode psychosis: a longitudinal network approach
Source: Transl Psychiatry. 2021 Nov 6;11:567. doi: 10.1038/s41398-021-01687-y (PMC8572227; doi:10.1038/s41398-021-01687-y)
Supplement: Supplementary file 3 — Figure 3. Baseline bootstrapped difference test between node strength [file 41398_2021_1687_MOESM3_ESM.pdf]

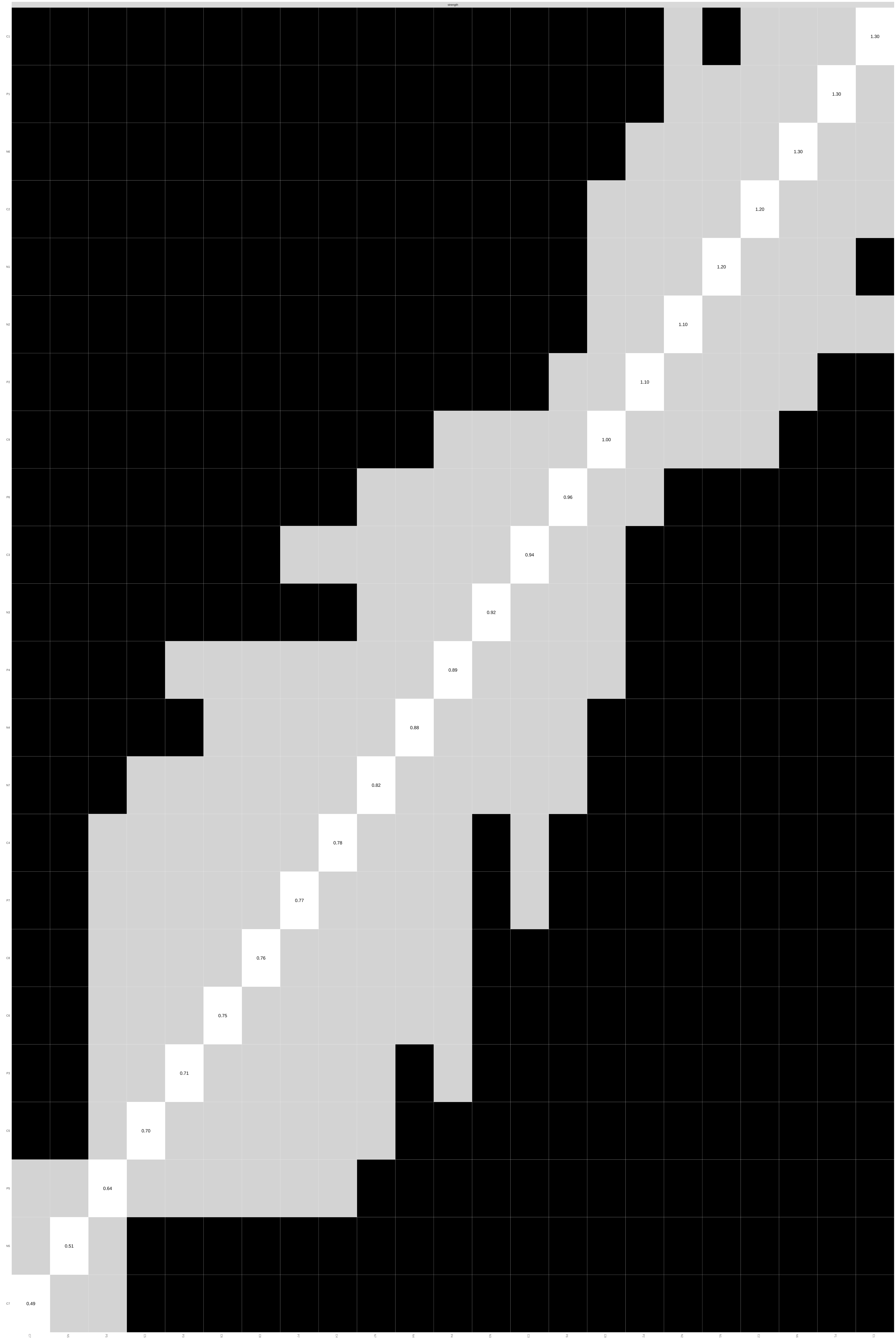
strength
C1
P1
N6
C2
N1
N2
P2
C9
P6
C3
N3
P4
N4
N7
C4
P7
C8
C6
P3
C5
P5
N5
C7
1.30
1.30
1.30
1.20
1.20
1.10
1.10
1.00
0.96
0.94
0.92
0.89
0.88
0.82
0.78
0.77
0.76
0.75
0.71
0.70
0.64
0.51
0.49
C7
N5
P5
C5
P3
C6
C8
P7
C4
N7
N4
P4
N3
C3
P6
C9
P2
N2
N1
C2
N6
P1
C1
